# Renal Replacement Therapy as a New Indicator of Voriconazole Clearance in a Population Pharmacokinetic Analysis of Critically Ill Patients

**DOI:** 10.3390/ph17060665

**Published:** 2024-05-22

**Authors:** Yuqiong Wang, Qinghua Ye, Pengmei Li, Linna Huang, Zhijiang Qi, Wenqian Chen, Qingyuan Zhan, Chen Wang

**Affiliations:** 1 Peking University China-Japan Friendship School of Clinical Medicine, Beijing 100029, China; 18703445333@163.com (Y.W.); cyh-birm@263.net (C.W.); 2 National Center for Respiratory Medicine, State Key Laboratory of Respiratory Health and Multimorbidity, National Clinical Research Center for Respiratory Diseases, Institute of Respiratory Medicine, Chinese Academy of Medical Sciences, Department of Pulmonary and Critical Care Medicine, Center of Respiratory Medicine, China-Japan Friendship Hospital, Beijing 100029, China; 18811176235@163.com (Q.Y.); xuehuaalice@126.com (L.H.); thymcglqi@163.com (Z.Q.); 3 Department of Pharmacy, China-Japan Friendship Hospital, Beijing 100029, China; lipengmei@yeah.net; 4 Chinese Academy of Medical Sciences and Peking Union Medical College, Beijing 100730, China

**Keywords:** voriconazole, pharmacokinetics/pharmacodynamics, critically ill, CRRT, ECMO

## Abstract

Aims: The pharmacokinetic (PK) profiles of voriconazole in intensive care unit (ICU) patients differ from that in other patients. We aimed to develop a population pharmacokinetic (PopPK) model to evaluate the effects of using extracorporeal membrane oxygenation (ECMO) and continuous renal replacement therapy (CRRT) and those of various biological covariates on the voriconazole PK profile. Methods: Modeling analyses of the PK parameters were conducted using the nonlinear mixed-effects modeling method (NONMEM) with a two-compartment model. Monte Carlo simulations (MCSs) were performed to observe the probability of target attainment (PTA) when receiving CRRT or not under different dosage regimens, different stratifications of quick C-reactive protein (qCRP), and different minimum inhibitory concentration (MIC) ranges. Results: A total of 408 critically ill patients with 746 voriconazole concentration–time data points were included in this study. A two-compartment population PK model with qCRP, CRRT, creatinine clearance rate (CL_CR_), platelets (PLT), and prothrombin time (PT) as fixed effects was developed using the NONMEM. Conclusions: We found that qCRP, CRRT, CL_CR_, PLT, and PT affected the voriconazole clearance. The most commonly used clinical regimen of 200 mg q12h was sufficient for the most common sensitive pathogens (MIC ≤ 0.25 mg/L), regardless of whether CRRT was performed and the level of qCRP. When the MIC was 0.5 mg/L, 200 mg q12h was insufficient only when the qCRP was <40 mg/L and CRRT was performed. When the MIC was ≥2 mg/L, a dose of 300 mg q12h could not achieve ≥ 90% PTA, necessitating the evaluation of a higher dose.

## 1. Introduction

Owing to unavoidable factors, patients in the intensive care unit (ICU) are more likely to receive long-term broad-spectrum antibiotics and glucocorticoids and invasive surgery (including extracorporeal membrane oxygenation (ECMO) catheterization, catheter placement, and hemodialysis), making them more susceptible to fungal infections [1]. Voriconazole is a new-generation triazole antifungal drug recommended as the first-line treatment against invasive aspergillosis by the Infectious Diseases Society of America (IDSA) [2], and it is also used as an alternative therapy for candidemia [3]. As such, voriconazole is widely used in ICU patients. The serum concentration of voriconazole can vary by up to 100-fold in individuals receiving the same dose [4] as it depends on several covariates, including age, weight, liver function, drug interactions, inflammation, genetic factors, and classical nonlinear pharmacokinetics (PKs) [5]. However, its therapeutic range is narrow; when the plasma trough concentration is too low (<1 mg/L), the therapeutic effect is poor, whereas supratherapeutic plasma trough concentrations (>5.5 mg/L) have been associated with an increased incidence of visual impairment, neurotoxicity, and hepatotoxicity [6].

The pathophysiological characteristics of the critically ill population differ from those of the general ward population; therefore, the PK profiles of voriconazole are significantly different in ICU patients [7]. Patients admitted to the ICU often show changes in fluid balance because of fluid therapy, capillary leakage, changes in plasma protein binding caused by hypoalbuminemia, and altered renal and hepatic function [7,8,9]. These changes are often accompanied by complex co-administered medications, systemic inflammation, bleeding, and transfusion, which can impact the drug clearance rate (CL) and apparent volume of distribution (Vd) [10]. 

Despite a number of studies evaluating voriconazole PKs in critically ill patients [11,12,13], these studies had small sample sizes, focused only on specific diseases, and lacked the simultaneous analysis of vital factors such as ECMO and continuous renal replacement therapy (CRRT). The frequency of ECMO and CRRT implementation in our ICU department is approximately 6% and 20%, respectively. Of note, the use of ECMO and CRRT is common in ICU patients, with some of them even using both machines concomitantly. Hence, discussing their effects on the voriconazole PK profile separately is unscientific. 

Voriconazole exposure is generally believed to be affected by ECMO; however, confirming this speculation is hard as literature is limited, with only five ex vitro studies [14,15,16,17,18], seven case reports [19,20,21,22,23,24,25], and two retrospective studies [26,27]. Moreover, while some studies have indicated that renal function has no effect on clearance [28], others have suggested that reduced renal function may lead to an increase in voriconazole plasma concentrations; however, no clear conclusions can be drawn because of the limited sample size or retrospective nature of these studies [12,29,30,31]. Similarly, although the overall PK of voriconazole is considered to be virtually unaffected by any mode of renal replacement therapy [32,33], one previous study indicated that continuous veno-venous hemofiltration (CVVH) with an ultrafiltration rate of 35 mL/(kg·h) may affect voriconazole clearance [34]. Some experts doubt whether even small amounts of voriconazole can be adsorbed onto the hemofilter membrane in the same way as onto the ECMO membrane; however, no relevant research has confirmed this hypothesis [35].

In view of the properties of voriconazole and the complex physiological changes and various operations in ICU patients, elucidating the PK characteristics of voriconazole in this special population has become challenging. Population pharmacokinetic (PopPK) modeling is widely used in the field because it helps to obtain the sources of PK variability [36]. Therefore, in this study, we developed a PopPK model to evaluate the factors influencing voriconazole PKs in critically ill patients. Monte Carlo simulations (MCS) were then performed to observe the probability of target attainment (PTA) when receiving CRRT or not under different dosage regimens, different stratifications of quick C-reactive protein (qCRP), and different minimum inhibitory concentration (MIC) ranges. To our knowledge, this is the largest PK study on voriconazole in ICU patients aimed at improving its dosing strategies for critically ill patients. 

## 2. Results

### 2.1. Baseline Characteristics

**Figure 1:**
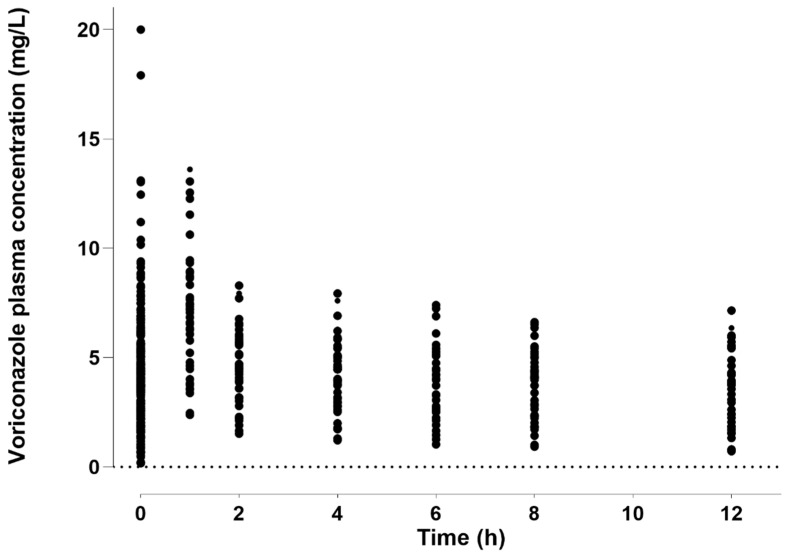
Concentration–time profile of voriconazole concentrations. Therapeutic drug monitoring (TDM) and PK study data are shown.

A total of 408 critically ill patients with 746 voriconazole concentration–time data points were included in this study. Among these, 287 concentration points were collected prospectively and continuously in 42 patients, whereas 459 trough concentrations were obtained retrospectively via routine therapeutic drug monitoring (TDM) in 366 patients. A total of 23 patients were excluded due to incomplete information or their use of a course of drugs that significantly affects voriconazole PKs. Figure 1 shows the time-dependent concentrations of voriconazole. The patient demographics are summarized in Table 1. Of the 408 participants, 287 (70.3%) were men. The mean age of patients was 64 years, while their mean weight was 65.3 kg. The patients showed considerable variability in blood biochemical parameters. During the sampling period, 85 patients received ECMO at 154 concentration points. CRRT was administered to 104 patients at 185 concentrations. As the study population consisted of patients in the respiratory ICU, each patient had either mild or severe lung infection. The most dominant voriconazole dosing regimen was 200 mg every 12 h (342 patients (83.8%)), with the remaining dosing regimens adjusted based on the TDM results.

### 2.2. Pharmacokinetic Model Building and Model Evaluation

The objective function values (OFVs) of the 1- and 2-compartment model are 1619.599 and 1334.118 respectively. A two-compartment model with first-order elimination adequately characterized voriconazole pharmacokinetics. The population estimates of the CL, central distribution volume (Vc), peripheral distribution volume (Vp), and intercompartmental clearance (Q) were 3.55 L/h (3.5%), 33.5 L (19.1%), 138 L (18.6%), and 52.8 L/h (15.9%), respectively. The inter-individual variability and the residual variability were described by the exponential model and the combined error model, respectively. 

In the forward selection procedure, the covariates qCRP, CRRT, creatinine clearance rate (CL_CR_), platelets (PLT), prothrombin time (PT), and aspartate transaminase (AST) were added to the parameter CL, with decreases in the OFV to 62.678, 23.757, 20.572, 18.755, 15.388, and 10.248, respectively. In the backward elimination steps, the increases in the OFV were 63.738, 34.945, 9.196, 18.755, 11.84, and 11.066, respectively. AST was removed from the final model because it had a poor relative standard error (RSE) (77%) and low estimate value (0.08). Therefore, the final model is:

$$\mathrm{CL}={CL}_{TV}\times {\left(\frac{qCRP}{73.6}\right)}^{-0.142}\times {\left(\frac{{CL}_{CR}}{71.8}\right)}^{0.218}\times 1.46^{\mathrm{CRRT}}\times {\left(\frac{PLT}{144}\right)}^{0.166}\times {\left(\frac{PT}{15}\right)}^{-0.875}\times e^{\eta_{CL}}$$

where CL_TV_ is the typical value of the total voriconazole CL. 

**Figure 2:**
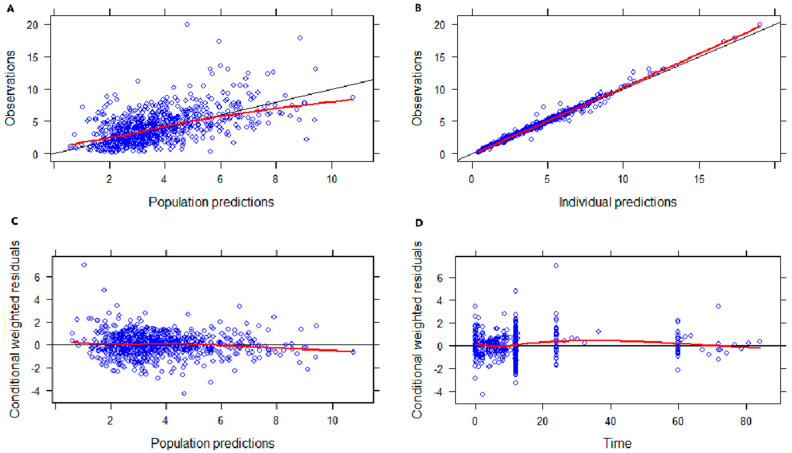
Goodness-of-fit plots of the final model. (**A**) Observed concentration (DV) versus population-predicted concentration (PRED). (**B**) DV versus individual−predicted concentration (IPRED). (**C**) Conditional weighted residuals (CWRES) versus PRED. (**D**) CWRES versus time.

The basic goodness-of-fit (GOF) plots presented in Figure 2 show the good predictive performance of the developed model. Both the population predictions and the individual predictions showed good agreement with the observations (Figure 2A,B). The conditional weighted residuals (CWRES) were uniformly distributed around zero with no trend, and most points were located within the accepted range (y = ±2) (Figure 2C,D). After inspection, no abnormal concentration points were found. 

The population parameter estimates obtained from the final PopPK model were close to the median bootstrap values and fell within the 95% CI calculated using the bootstrap method, indicating that the final model was stable and robust (Table 2). The PK parameters grouped by ECMO and CRRT were listed in Table 3 and Table 4, respectively. The CL increased in the CRRT group (*p* < 0.05), and there was no significant difference in other PK parameters. The PK parameters grouped by the route of administration were listed in Table S1, and there was no significant difference in the PK parameters.

**Figure 3:**
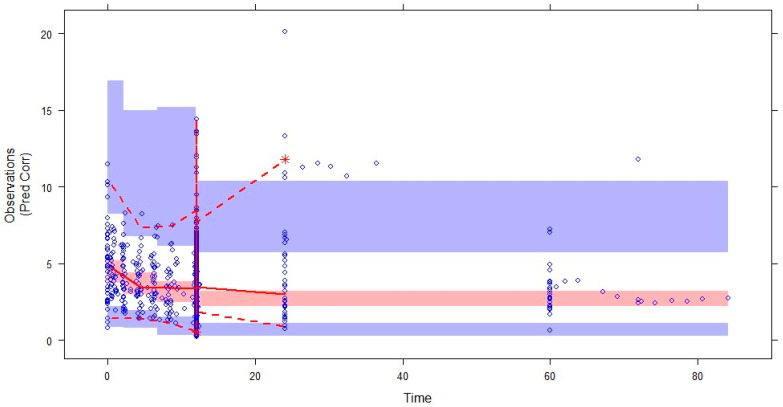
Prediction-corrected VPC (pcVPC) for the final pharmacokinetic model of voriconazole. The blue circles represent the observed data. The middle solid, lower dashed, and upper dashed lines represent the median, 5th, and 95th percentiles for the observed data, respectively. The shaded areas represent a 95% CI for the simulated predicted median, 5th, and 95th percentiles constructed from simulated datasets of individuals from the original data.

The prediction-corrected visual predictive check (pcVPC) plots based on 1000 simulations on the data is shown in Figure 3. Most of the observed data were within the 95% CIs of the 5th, 50th, and 95th percentiles of the simulated data (shaded areas), showing good performance in predicting the plasma concentrations of voriconazole. 

### 2.3. Simulations and the PTA 

Table 5 shows the achievable simulated PTAs of critically ill patients under different dosage regimens when combined with the common clinical MIC stratification, qCRP stratification, and whether CRRT was performed. Overall, 144 different clinical scenarios were simulated. Standardized values of the covariates included in the final model were taken for each individual. The simulation results showed that the PTA value during CRRT decreased compared with that without CRRT. When the patient underwent CRRT, with an MIC ≤ 0.25 mg/L, all simulation plans achieved a ≥90% PTA; with an MIC of 0.5 mg/L, a regimen of at least 200 mg q12h was required to achieve a ≥90% PTA (except when qCRP was 40 mg/L, where a dose of 250 mg q12h was required); with an MIC of 1 mg/L, none of the simulation plans achieved a ≥90% PTA, necessitating the evaluation of a higher dose.

## 3. Discussion

Voriconazole is a key life-saving drug in the treatment of invasive aspergillosis, and its blood concentration is directly related to the prognosis of the disease. However, patients in the ICU are often treated with CRRT and ECMO, which are considered to result in substandard drug concentrations. In this study, we collected extensive data on voriconazole TDM among respiratory ICU patients, as well as prospective intensive sampling data, which allowed us to investigate the influence of multiple covariates on voriconazole PK parameters. This is the first study to simultaneously explore the effects of ECMO, CRRT, and various physiological and biochemical factors on the PK/pharmacodynamic (PD) profile of voriconazole in ICU patients, and it is the largest study on ICU patients to date. As such, this study revealed several novel findings. 

### 3.1. PK Parameters of This Model 

A strong correlation has been demonstrated between plasma and lung epithelial lining fluid (ELF) voriconazole concentrations [37,38]. For practical reasons, using plasma instead of ELF concentrations is preferred. Previous literature on the administration of voriconazole in critically ill patients is limited. Our findings suggested that a two-compartment model with first-order elimination is optimal for modeling voriconazole PK data in critically ill patients. The estimated Vc and Vp in our study were approximately the same as those in a prospective study of 33 ICU patients treated with intravenous voriconazole (28.2 L and 157.3 L, respectively) [39]. According to this model, voriconazole CL was 3.55 L/h, in agreement with the range of 2.88–4.28 L/h reported in other studies [11,29,40]. 

### 3.2. CRRT Affects Voriconazole CL

Our observation that CRRT increased voriconazole CL is important because previous data on the elimination of this moderate plasma protein-bound drug in patients receiving CRRT are limited. Conventional wisdom holds that renal and extracorporeal clearance only account for 1–15% of total voriconazole CL, that the overall PKs of voriconazole is virtually unaffected by renal replacement regardless of the mode, and that voriconazole dose adjustment is not necessary [32]. For example, a study including six patients with CVVH concluded that CVVH had no clinical significance on voriconazole CL [33]. Likewise, two case reports on 10 critically ill patients requiring continuous veno-venous hemofiltration (CVVHDF) found that voriconazole dose adjustment was not required [32,41]. In contrast, Quintard et al. [34] studied the PKs of voriconazole in a critically ill patient with anuria who was administered 4 mg/kg voriconazole and under high-volume CVVH at an ultrafiltration rate of 35 mL/kg/h, and found that CVVH helped eliminate 21% of the drug. However, the number of participants in these studies was too small to ensure scientific validity. In contrast, 104 people with 186 concentration points included in our study underwent the CVVH procedure, making them the largest research population on the relationship between CRRT and voriconazole PKs. 

Theoretically, drugs with a molecular weight > 5000 Da, high protein binding capacity (>80%), and a large Vd (>1 L/kg) are the least likely to be removed by CRRT [42]. Voriconazole has a molecular weight of 349.31 Da and is widely distributed throughout the body, with 58% of it being bound to plasma proteins, complicating the determination of its clearance. In our ICU, bedside nurses performed CRRT uniformly, and all included patients underwent the CVVH procedure. Except in special circumstances, the blood flow rate was set to approximately 120–150 mL/min, the replacement fluid speed was set to approximately 25–30 mL/(kg·h), and the “predilution” method was adopted. All machines were obtained from Fresenius, and all consumables were purchased together. The membranes employed for CVVH typically contain small pores, easily eliminating molecules smaller than 500 Da. Therefore, we speculated that voriconazole may have been filtered out through the pores. In addition, 50.8% of the CRRT population had hypoalbuminemia (<35 g/L) [43], which resulted in a higher proportion of unbound drugs and more drugs being cleared by the liver and CRRT. Notably, a previous study using an integrated dialysis pharmacometric model suggested that a small amount of voriconazole may be adsorbed onto the blood filtration membrane [35]. Thus, determining whether voriconazole is adsorbed onto the hemofilter membrane in the same way as onto the ECMO membrane [14,15] may be important for determining the increase in voriconazole clearance caused by CRRT. 

### 3.3. Voriconazole CL Increases When the CL_CR_ Increases

Surprisingly, we found that voriconazole CL increased with increasing CL_CR_, suggesting that renal function may indeed affect voriconazole CL. By contrast, less than 2% of an oral or intravenous dose of voriconazole is believed to be excreted unchanged in the urine; that is, voriconazole CL is not affected by renal function. However, a previous study [12] reported higher voriconazole plasma concentrations in patients with moderate renal impairment (CL_CR_ 40–55 mL/min) than in those with normal renal function (CL_CR_ ≥ 60 mL/min) following the administration of 320 mg and 240 mg doses. Similar findings have been reported using multiple linear regression analyses [30,31,44,45]. In a prospective PK study involving 105 kidney transplant recipients (342 concentrations), the reported voriconazole CL was 2.88 L/h, and the authors speculated that the low CL might be attributed to the unrecovered kidney function [29]. Another possible explanation is that the CL_CR_ value, which can indirectly reflect the clearing effect of CRRT, could be affected by CRRT factors and therefore reflected in the final model.

### 3.4. Voriconazole CL Increases When the Platelet Count Increases

Unexpectedly, we found a positive correlation between platelet count and voriconazole CL. A review of the literature revealed similar findings in other studies. For example, Tang et al. included 166 samples extracted from 57 patients with liver dysfunction and found that a low platelet count was associated with a significant reduction in voriconazole CL [46]. Another study included 51 patients with liver dysfunction and reached similar conclusions [47]. In patients that underwent kidney transplantation [48], a one-unit increase in platelet count was associated with a 0.004 mg/L decrease in voriconazole trough concentration. Nevertheless, none of the above-mentioned studies explored the reasons for this finding. Platelet count is related to liver function [49]. When liver function declines, the associated portal hypertension and decreased thrombopoietin levels lead to decreased platelet count [50]. Therefore, the platelet count observed in our study may just be a reflection of liver function. The scatter diagram (Supplementary Figure S1A,B) between PLT and AST, as well as between PLT and total bilirubin, confirmed this hypothesis. Therefore, further experiments are required to elucidate the potential mechanism and to confirm whether PLT are innocent bystanders or active players. 

### 3.5. Voriconazole CL Increases When qCRP Decreases

Our study found that an increase in qCRP was related to a decrease in voriconazole CL, which has been widely confirmed in previous studies [39,51,52,53,54,55,56]. This is attributed to the fact that, in an inflammatory state, inflammatory mediators can bind to cytokine and toll-like receptor 4 receptors on the cell membrane and regulate the expression of transporters and drug-related metabolic enzymes through the NF-κB signaling pathway [57]. These findings suggested that metabolizing enzymes, including cytochrome P450 (CYP) isoenzymes, are downregulated by inflammatory cytokines, resulting in a decrease in voriconazole CL [56,58]. Furthermore, the inflammatory status may modulate polymorphisms in PK-related genes, which may influence the metabolic pathway from voriconazole to voriconazole N-oxide [54]. 

### 3.6. Voriconazole CL Increases When PT Decrease

In our study, the increase in PT was associated with an increase in the voriconazole concentration. To date, no other PK studies have reported such a finding. Although no collinearity was observed when the covariates were included in the model, we suspect that a correlation exists between PT and liver function. PT, which are mainly metabolized in the liver through CYP isozymes [59], are considered a reliable marker of liver protein synthesis and, therefore, of the liver functional reserve [60]. The scatter diagram (Supplementary Figure S2A,B) between PT and AST, as well as between PT and total bilirubin, confirmed this hypothesis.

### 3.7. No Effect of ECMO on Voriconazole CL Was Observed

We did not observe any effect of ECMO on voriconazole CL, despite previous in vitro studies [14,15,16,17,18] and one retrospective study [26] reporting that ECMO affects voriconazole PKs. Nevertheless, the largest study to date, a retrospective study from eight centers in four countries (69 patients, 337 samples), suggested that ECMO had no significant effect on voriconazole exposure [27], consistent with our results. One study mentioned binding-site saturation to explain the fluctuations in voriconazole concentrations in ECMO patients [21]. However, the ECMO population in our study was small (85 patients), which may have obscured the impact of ECMO on voriconazole; thus, a larger population may be needed to resolve this controversial issue in the future.

### 3.8. Limitations

Our study had several limitations. First, the study population was limited to a single-center respiratory ICU, with pulmonary infection being the main diagnosis; therefore, the results may not necessarily reflect those of other ICU patients. Second, other factors, such as diet and CYP genotyping, were not tested in this study but may have an impact on voriconazole PK. Future studies should also include measurements of dialysis specimens to more accurately assess the potential impact of CRRT on voriconazole CL. Finally, in the simulation based on literature data from healthy volunteers, the protein binding rate was fixed at 58%. However, in severely ill patients, due to generally low protein levels, the protein binding rate may be different, thus limiting the applicability of models and simulations.

## 4. Materials and Methods

### 4.1. Study Design and Ethics

This study was conducted in a 22-bed respiratory ICU at a 1600-bed teaching tertiary hospital in Beijing. In this department, voriconazole is used approximately 15% of the time for various reasons (prophylactic or therapeutic). We combined prospective intensive sampling and retrospective trough concentration monitoring with routine TDM. This study included patients who were admitted to the respiratory ICU from January 2017 to December 2023 and received intravenous or oral/nasogastric voriconazole (200 mg, Pfizer Pharmaceuticals Ltd., Kalamazoo, MI, USA). The exclusion criteria were as follows: (1) age < 18 years; (2) pregnancy; (3) lack of important dosing information or clinical data; and (4) concomitant use of drugs known to significantly affect the PKs of voriconazole, such as rifampicin, rifabutin, phenytoin, phenobarbital, or carbamazepine. This clinical study was approved by the Ethics Committee of the China–Japan Friendship Hospital (2022-KY-113-1), and written informed consent was obtained from all prospective participants involved in the study. Considering ethical requirements, we only collected 1 mL of blood for testing.

### 4.2. Drug Regimens and Clinical Data

Voriconazole was administered as recommended by clinicians, whether prophylactically, empirically, or based on microbiological outcomes. We did not interfere with the dose or frequency of drug administration. Retrospective trough concentrations were monitored when the doctor deemed it necessary (usually 5–7 d after administration), and prospective samples were collected when the drug concentration reached a stable state. The definition of the voriconazole stable-state trough concentration is as follows: the trough concentration measured after voriconazole loading-dose treatment for >3 d or without loading-dose treatment for >5 d [61,62,63]. 

Prospective blood samples (1 mL) were collected from patients using purple blood collection tubes anticoagulated with ethylenediaminetetraacetic acid via an arterial catheter before the start of infusion, at the end of infusion (with voriconazole dissolved in 50 mL or 100 mL of solvent, typically completing the infusion within 30–60 min), and at 2, 4, 6, 8, and 12 h after infusion. The specimens were centrifuged at 2000× *g* for 5 min, transferred into polypropylene tubes, and frozen at −80 °C until use. The TDM trough concentration was measured by a nurse 30 min before the next dose and immediately sent to the Department of Pharmacy at our hospital for testing.

For each patient, demographic data (age, sex, weight, height, body mass index (BMI)), laboratory test results (routine blood examination, alanine transaminase (ALT), AST, bilirubin, albumin, creatinine, CL_CR_, estimated glomerular filtration rate (eGFR), urea, qCRP), the Acute Physiology and Chronic Health Evaluation (APACHE) II score, the Sequential Organ Failure Assessment (SOFA) score, CRRT condition, ECMO condition, medication records (dose, time, and frequency), concomitant medications (glucocorticoids and proton-pump inhibitors), and clinical outcomes were recorded. Of note, CL_CR_ was calculated using the Cockcroft–Gault equation.

### 4.3. Drug Assay

The concentration of voriconazole was analyzed using an ultrahigh-performance liquid chromatography–tandem mass spectrometry (UPLC-MS) method previously validated in our laboratory [26]. Liquid chromatography was performed with a Waters Acquity UPLC system with an Acquity UPLC^©^ BEH-C_18_ column (50 mm × 2.1 mm, 1.7 μm) at 40 °C. The mobile phases were pumped at a flow rate of 0.2 mL/min and consisted of 2 mmol/L ammonium acetate and 0.1% formic acid (mobile phase A) and acetonitrile containing 0.1% formic acid (mobile phase B). A Waters Quattro Premier XE triple–quadrupole mass spectrometer was used to detect the analytes. Quantification was accomplished via electrospray ionization in the positive ion mode with multiple-reaction monitoring. The lower limit of quantification was 0.097 mg/L for the analytes. The calibration curves were linear over a range of 0.097–12.500 mg/L. The intra- and inter-day precision was less than 7%. The accuracy, extraction recovery, matrix effect, and intra- and inter-assay precision all met the requirements for quantitative analysis of in vivo concentrations. 

### 4.4. Model Selection

#### 4.4.1. Structural Model 

The modeling analysis and calculation of the pharmacokinetic parameters were conducted using the nonlinear mixed-effects modeling method (NONMEM, version 7.2.0, ICON Development Solutions, Ellicott City, MD, USA) using a two-compartment model with linear elimination kinetics. The first-order conditional estimation method was applied to all model runs. The CL, Vd, the area under the drug plasma concentration–time curve over 24 h of voriconazole (AUC_24_), and bioavailability (F) were characterized and estimated. The absorption rate constant (Ka) was fixed to a value of 1.2/h, as reported elsewhere [64,65]. We used the Akaike information criteria (AIC) to compare the models, and the model with the lowest AIC value was considered the best. At the same time, we also consider graphic criteria such as GOF plots and pcVPC plots. 

#### 4.4.2. Statistical Model

Inter-individual variations in voriconazole pharmacokinetics were modeled exponentially: P_ij_ = P_pop_ × exp(η_ij_), where P_ij_ is the j-th pharmacokinetic parameter estimation of the i-th individual, P_pop_ is the population typical value of the j-th parameter, and η_ij_ is an inter-individual random variable distributed with a mean of 0 and a variance of ω^2^. 

Residual variability was evaluated by the combined error model: C_obs_ = C_pred_ × (1 + ε) + ε’, where C_obs_ and C_pred_ are the observed and predicted concentrations, while ε and ε’ are random variables distributed with a mean of 0 and variances of σ^2^ and σ’^2^, respectively. 

#### 4.4.3. Covariate Model 

After base model development, the effect of the potential covariates on voriconazole PK parameters were studied using a stepwise forward selection and backward elimination steps. Preliminary inspections were made on the potential impact of individual covariates on voriconazole PK parameters based on scatterplots (continuous variables) and boxplots (categorical variables) of η values against covariates. Covariates associated with a significant decrease in the OFV (OFV defined as −2 times the log-likelihood) of greater than 3.84 (*p* < 0.05, χ^2^ distribution with 1 degree of freedom) were added to the base model. Then, covariates that resulted in a significant increase in the OFV of at least 7.88 (*p* < 0.005, χ^2^ distribution with 1 degree of freedom) were retained in the final model during the backward deletion. Additional criteria for evaluating the covariates included were a reduction in unexplained inter-individual variability, diagnostic plots of the weighted residuals, and the GOF. Only biologically plausible covariates could be included in the final model. 

The continuous covariates examined were age, weight, height, BMI, body surface area, white blood cell count, neutrophil count, lymphocyte count, hemoglobin, PLT, qCRP, coagulation indicators, AST, ALT, bilirubin, albumin, creatinine, CL_CR_, eGFR, urea, the APACHE II score, and the SOFA score. The categorical covariates examined were sex, co-medications such as proton pump inhibitors and glucocorticoids, whether to perform an ECMO, and whether to perform a CRRT. 

### 4.5. Model Validation

The model was validated by GOF analyses, the nonparametric bootstrap method and pcVPC. The GOF analyses consisted of four plots as follows: the observed concentration (DV) vs. individual-predicted concentration (IPRED), DV vs. the population-predicted concentration (PRED), the CWRES vs. time, and CWRES vs. PRED graphs. 

The nonparametric bootstrap procedure was conducted using 1000 randomly re-sampled data to evaluate the stability of the final model. The parameters (median and 95% CI) obtained from the bootstrap analysis were compared with the estimates of the final model. 

Meanwhile, the pcVPC method was also used to graphically evaluate the adequacy of fitting. The data set was simulated 1000 times, and the simulated concentrations (5th, 50th, and 95th percentiles) were compared with the observed data.

### 4.6. Monte Carlo Simulations

We used the free (unbound to plasma proteins) area under the concentration–time curve from 0 to 24 h (ƒAUC_24_) divided by the MIC (ƒAUC_24_/MIC) > 25 as the PK/PD index (64). A value of 58% protein binding in human plasma was used to simulate *f*AUC_24_ (65). We then used MCS (n = 1000) to evaluate the impact of CRRT and qCRP on the probability of achieving voriconazole PK/PD targets under different dosing regimens. The most common MICs for clinical isolates of *Aspergillus* and *Candida* in our hospital, as certified by microbiology laboratory physicians, were used as PD factors. The MCS results were expressed as the PTA, with a PTA value of >90% considered an optimal empirical dosing regimen.

The following three dosages were selected for the simulation: (1) 200 mg every 12 h by intravenous infusion; (2) 250 mg every 12 h by intravenous infusion; and (3) 300 mg every 12 h by intravenous infusion. All simulated infusion times were 1 h. The simulated MICs were 0.016, 0.032, 0.25, 0.5, 1, 2, 8, and 16 mg/L for *Aspergillus* or *Candida* infections.

### 4.7. Statistical Analysis

Statistical analyses were performed with SPSS 29.0, and figures were drawn using GraphPad Prism 10.0. Continuous variables were expressed as the mean ± SD or median and interquartile range (IQR). Categorical variables are presented in terms of frequency (%). The Student’s *t* test and the Mann–Whitney *U* test were used to compare continuous variables and the chi-square test and Fisher’s exact test were used to compare categorical variables. Statistical significance was defined as a *p* < 0.05.

## 5. Conclusions

In this study, we established a PopPK model of voriconazole and evaluated the effects of ECMO, CRRT, and various physiological and biochemical factors on PK parameters. We found that the qCRP, CRRT, CL_CR_, PLT, and PT affected the PK parameter CL. The most commonly used clinical regimen of 200 mg q12h was sufficient against the most common sensitive pathogens (MIC ≤ 0.25 mg/L) [66], regardless of whether CRRT was performed and the level of qCRP. When the MIC was 0.5 mg/L, 200 mg q12h was insufficient only when the qCRP was <40 mg/L and CRRT was performed. When the MIC was 1 mg/L, a dose of 200 mg q12h was insufficient. When the MIC was ≥2 mg/L, a dose of 300 mg q12h could not achieve a ≥90% PTA, necessitating the evaluation of a higher dose.

## Figures and Tables

**Table 1:** Characteristics of patients included in the study.

Characteristic	Value ^a^
Age (years)	66 (57, 73)
Sex (male/female)	287/121
Weight (kg)	65 (55, 75)
Height (cm)	169 (162, 173)
Body mass index (kg/m^2^)	23.2 (20.3, 25.7)
APACHE II score on the day of blood collection	19.0 (14.0, 25.0)
SOFA score on the day of blood collection	7.0 (4.0, 10.0)
White blood cell count, 10^9^/L, median (IQR)	9.9 (6.7, 14.2)
Neutrophilic granulocyte count, 10^9^/L	8.4(5.4, 12.4)
Lymphocyte count, 10^9^/L	0.8 (0.5, 1.2)
Hemoglobin, 10^12^/L	85.0 (75.0, 100.8)
Platelet, 10^9^/L	150.5 (88.0, 223.8)
ALT (U/L)	27.0 (16.0, 51.0)
AST (U/L)	36.0 (23.0, 61.0)
Total bilirubin (µmol/L)	12.9 (8.1, 24.2)
Direct bilirubin (µmol/L)	5.7 (2.9, 11.9)
Albumin (mg/dL)	34.0 (30.3, 38.0)
Blood urea (mmol/L)	78.5 (54.4, 126.0)
SCR (μmol/L)	78.5 (54.4, 126.0)
CL_CR_ (mL/min)	68.5 (45.5, 102.5)
qCRP (mg/L)	73.6 (30.0, 160.0)
Procalcitonin (ug/L)	0.4 (0.2, 1.5)
Use of proton pump inhibitors (%)	353 (70.5)
Use of glucocorticoid (%)	197 (39.3)
ECMO (yes/no)	108/393
CRRT (yes/no)	122/379
Administration on day of PK sampling	
Intravenous infusion (%)	278 (68.1)
Oral (%)	45 (11.0)
Nasogastric (%)	85 (20.8)
No. (%) receiving voriconazole	
200 mg q12h	342 (83.8)
150 mg q12h	16 (3.9)
100 mg q12h	9 (2.2)
200 mg qm, 100 mg qn ^b^	7 (1.7)
Others	34 (8.3)

^a^ The median and interquartile range (IQR) are shown for continuous variables; the proportion is shown for categorical variables. These 408 patients generated a total of 501 on-machine occasions. Except for age, sex, weight, height, body mass index (BMI), dosing method and dosage, we used 501 as the base number for all statistics. ^b^ qm, every morning; qn, every night. Abbreviations: ALT, alanine transaminase; APACHE, Acute Physiology and Chronic Health Evaluation; AST, aspartate transaminase; CL_CR_, creatinine clearance; CRRT, continuous renal replacement therapy; ECMO, extracorporeal membrane oxygenation; qCRP, quick C-reactive protein; SCR, serum creatinine concentration; SOFA, Sequential Organ Failure Assessment.

**Table 2:** Population PK parameters of the final model.

	2-Compartment Model		Bootstrap (n = 1000)	
Parameter	Estimate	RSE (%)	Median	95% CI
Fixed effects				
CL (L/h)	3.55	3.5	3.55	3.33–3.77
Vc (L)	33.50	19.1	33.11	22.70–43.38
Vp (L)	138.00	18.6	142.45	107.88–183.39
Q (L/h)	52.80	15.9	53.05	41.34–70.24
Ka (/h)	1.20 (fixed)	-	1.20 (fixed)	-
F	0.835	5.8	0.83	0.75–0.93
θ_qCRP_CL_	0.142	14.6	0.14	0.10–0.19
θ_CRRT_CL_	1.46	5.9	1.46	1.29–1.65
θ_PLT_CL_	0.166	25.2	0.16	0.10–0.24
θ_PT_CL_	0.875	23.2	0.87	0.48–1.44
θ_CLCR_CL_	0.218	15.6	0.22	0.14–0.30
Random effects				
Inter-individual variability (% CV)				
CL (L/h)	49.80	4.4	49.29	45.05–53.31
Vc (L)	66.70	26.6	66.65	47.20–90.25
Vp (L)	81.70	21.8	78.39	46.57–115.06
Q (L/h)	0 (fixed)	-	0 (fixed)	-
Residual error (%CV if proportional, SD if additive)				
Additive (mg/L)	0.192	28.1	0.20	0.04–0.33
Proportional	8.9	9.5	8.8	3.78–12.19

Abbreviations: %CV, percent coefficient of variation; CI, confidence interval; CL, clearance; F, bioavailability; Ka, absorption rate constant; Q, intercompartmental clearance; RSE, relative standard error; SD, standard deviation; Vc, volume of distribution in the central compartment; Vp, volume of distribution in the peripheral compartment.

**Table 3:** Pharmacokinetic parameters grouped by CRRT.

	All (n = 501)	CRRT (n = 122)	Non-CRRT (n = 379)	*p*
CL (L/h)	3.78 (4.31–4.78)	3.99 (2.99–6.35)	3.73 (2.64–5.49)	0.027
Vc (L)	33.50 (32.84–33.85)	33.38 (32.52–34.06)	33.50 (32.62–34.00)	0.821
Vp (L)	138.34 (130.66–136.18)	137.15 (127.18–145.88)	138.38 (125.23–144.53)	0.655
AUC_24_ (mg·h/L)	90.20 (57.30–128.00)	87.90 (52.50–119.00)	91.50 (60.70–131.00)	0.114
Cmin (mg/L)	3.62 (1.92–5.33)	3.19 (1.73–5.03)	3.70 (2.03–5.45)	0.110
T1/2β (h)	6.21 (3.91–8.82)	5.87 (3.70–8.43)	6.34 (4.05–9.01)	0.068

Note: Data are shown as the median and interquartile range (IQR) for each parameter. The *p* value between the CRRT group and non-CRRT group was calculated. These 408 patients generated a total of 501 on-machine occasions. Abbreviations: AUC_24:_ the area under the drug plasma concentration–time curve over 24 h of voriconazole; CL, clearance; Cmin, trough plasma concentration; CRRT: continuous renal replacement therapy; T1/2β, elimination half-life; Vc, central distribution volume; Vp, peripheral distribution volume.

**Table 4:** Pharmacokinetic parameters grouped by ECMO.

	All (n = 501)	ECMO (n = 122)	Non-ECMO (n = 379)	*p*
CL (L/h)	3.78 (4.31–4.78)	3.60 (2.75–5.46)	3.79 (2.66–5.86)	0.929
Vc (L)	33.50 (32.84–33.85)	33.48 (32.11–33.95)	33.50 (32.67–34.01)	0.637
Vp (L)	138.34 (130.66–136.18)	138.11 (119.84–144.86)	138.38 (126.65–144.97)	0.789
AUC_24_ (mg·h/L)	90.20 (57.30–128.00)	93.90 (60.40–123.00)	89.70 (57.20–130.00)	0.943
Cmin (mg/L)	3.62 (1.92–5.33)	3.62 (2.06–5.29)	3.62 (1.90–5.36)	0.935
T1/2β (h)	6.21 (3.91–8.82)	6.51 (4.11–8.59)	6.15 (3.88–9.02)	0.898

Note: Data are shown as the median and interquartile range (IQR) for each parameter. The *p* value between the CRRT group and non-CRRT group was calculated. These 408 patients generated a total of 501 on-machine occasions. Abbreviations: AUC_24_: the area under the drug plasma concentration–time curve over 24 h of voriconazole; CL, clearance; Cmin, trough plasma concentration; ECMO: extracorporeal membrane oxygenation; T1/2β, elimination half-life; Vc, central distribution volume; Vp, peripheral distribution volume.

**Table 5:** Probability of target attainment (PTA) for CRRT, qCRP-based voriconazole regimens according to Monte Carlo simulations.

Dose	qCRP (mg/L)	CRRT	MIC (mg/L)							
			0.016	0.032	0.25	0.5	1	2	8	16
200 mg q12h	40	Yes	100	100	97.1	81	47.1	9	0	0
		No	100	100	99.5	93.6	74.3	35.3	0	0
	80	Yes	100	100	99.5	94.3	55.2	11	0	0
		No	100	100	99.5	99.1	83.6	35.5	0	0
	160	Yes	100	100	100	96.2	64.3	15.8	0	0
		No	100	100	100	99.6	87.5	44.6	0	0
250 mg q12h	40	Yes	100	100	100	96.5	65.8	17	0	0
		No	100	100	100	99.7	88.7	45.9	0	0
	80	Yes	100	100	100	97.6	71.5	21.8	0	0
		No	100	100	100	100	91.7	54.3	0	0
	160	Yes	100	100	100	98.4	77.4	28.4	0	0
		No	100	100	100	100	94.5	60.1	0	0
300 mg q12h	40	Yes	100	100	100	98	76.1	27.4	0	0
		No	100	100	100	100	94	59.3	0	0
	80	Yes	100	100	100	98.8	83.7	34.7	0	0
		No	100	100	100	100	96.4	67.7	0	0
	160	Yes	100	100	100	99.3	88.2	41.5	0	0
		No	100	100	100	100	97.8	75.1	0	0

Abbreviations: CRRT: continuous renal replacement therapy; MIC: minimum inhibitory concentration; qCRP: quick C-reactive protein.

## Data Availability

The datasets used and/or analyzed during the current study are available from the corresponding author upon reasonable request.

## Supplementary Materials

The following supporting information can be downloaded at: https://www.mdpi.com/article/10.3390/ph17060665/s1, Figure S1: A. The scatter diagram between PLT and AST; B. The scatter diagram between PLT and total bilirubin. Figure S2: A. The scatter diagram between PT and AST; B. The scatter diagram between PT and total bilirubin. Table S1. PK parameters grouped according to the route of administration.
