# Supplementary material for: Renal Replacement Therapy as a New Indicator of Voriconazole Clearance in a Population Pharmacokinetic Analysis of Critically Ill Patients
Source: Pharmaceuticals (Basel). 2024 May 22;17(6):665. doi: 10.3390/ph17060665 (PMC11206427; doi:10.3390/ph17060665)
Supplement: Supplementary file 1 [file pharmaceuticals-17-00665-s001.zip › Table S1.pdf]

Table S1. PK parameters grouped according to the route of administration

	All (n=501)	Oral/nasogastric feeding (n=130)	Intravenous administration (n=278)	*p*
CL (liters/h)	3.78 (4.31-4.78)	4.01 (2.57-6.36)	3.72 (2.68-5.69)	0.424
Vc (liters)	33.50 (32.84-33.85)	33.58 (32.27-33.96)	33.48 (32.68-34.01)	0.908
Vp (liters)	138.34 (130.66-136.18)	138.78 (120.66-144.15)	138.27 (127.00-145.33)	0.500
AUC_{24} (mg•h/L)	90.14 (97.90-109.82)	83.22 (47.26 -130.84)	92.42 (62.48-126.26)	0.109

Note: Data are shown with median and interquartile range (IQR) for each parameter. *p* value was calculated between the Oral/nasogastric feeding group and Intravenous administration group. These 408 patients generated a total of 501 on-machine occasions.

Abbreviations: $AUC_{24:}$ the area under drug plasma concentration-time curve over 24 h of voriconazole; CL, clearance; Vc, central distributionvolume; Vp, peripheral distribution volume.
