# Supplementary figures and images for: Renal Replacement Therapy as a New Indicator of Voriconazole Clearance in a Population Pharmacokinetic Analysis of Critically Ill Patients
Source: Pharmaceuticals (Basel). 2024 May 22;17(6):665. doi: 10.3390/ph17060665 (PMC11206427; doi:10.3390/ph17060665)

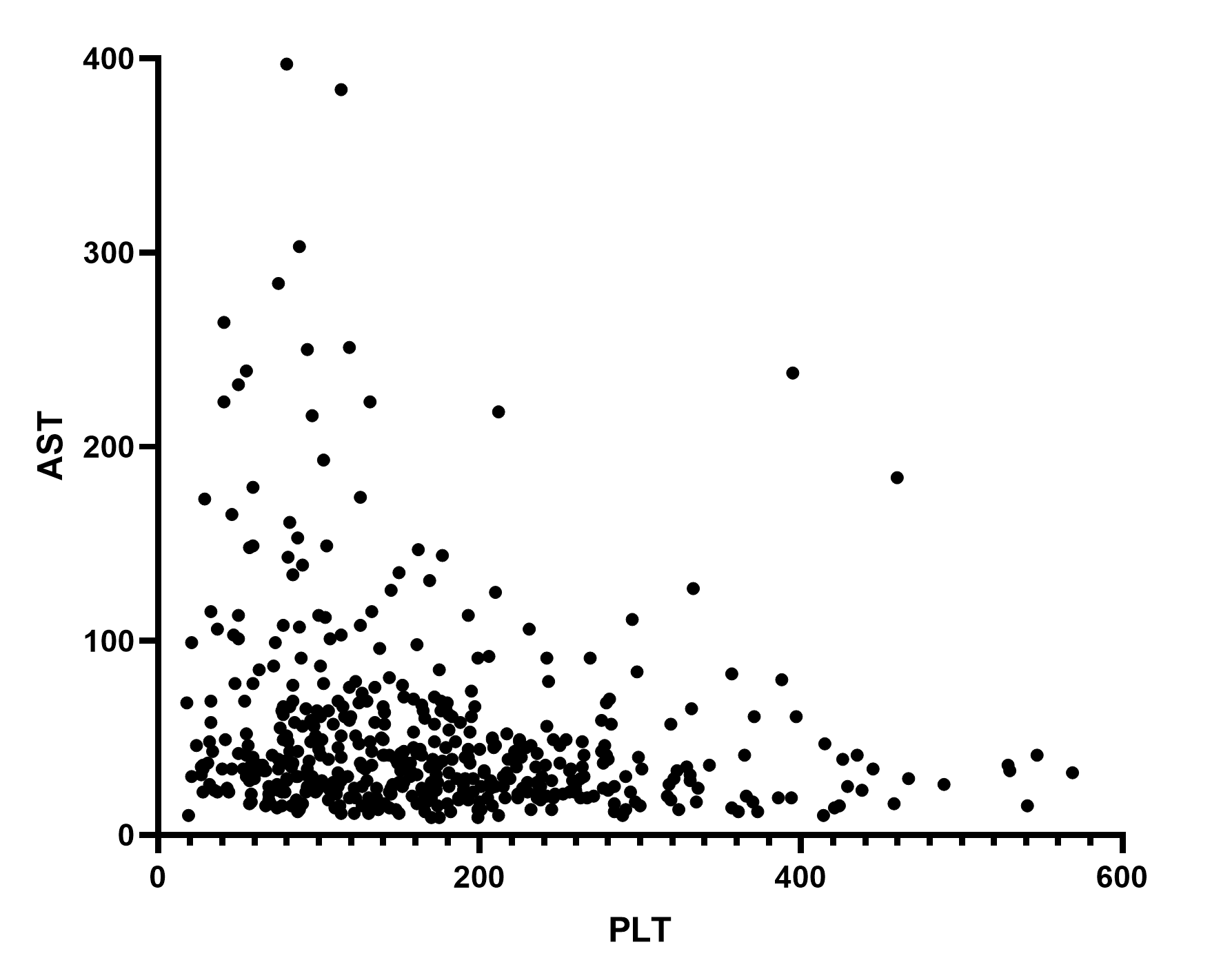

Supplement: Supplementary file 1 [file pharmaceuticals-17-00665-s001.zip › Figure S1A.png]

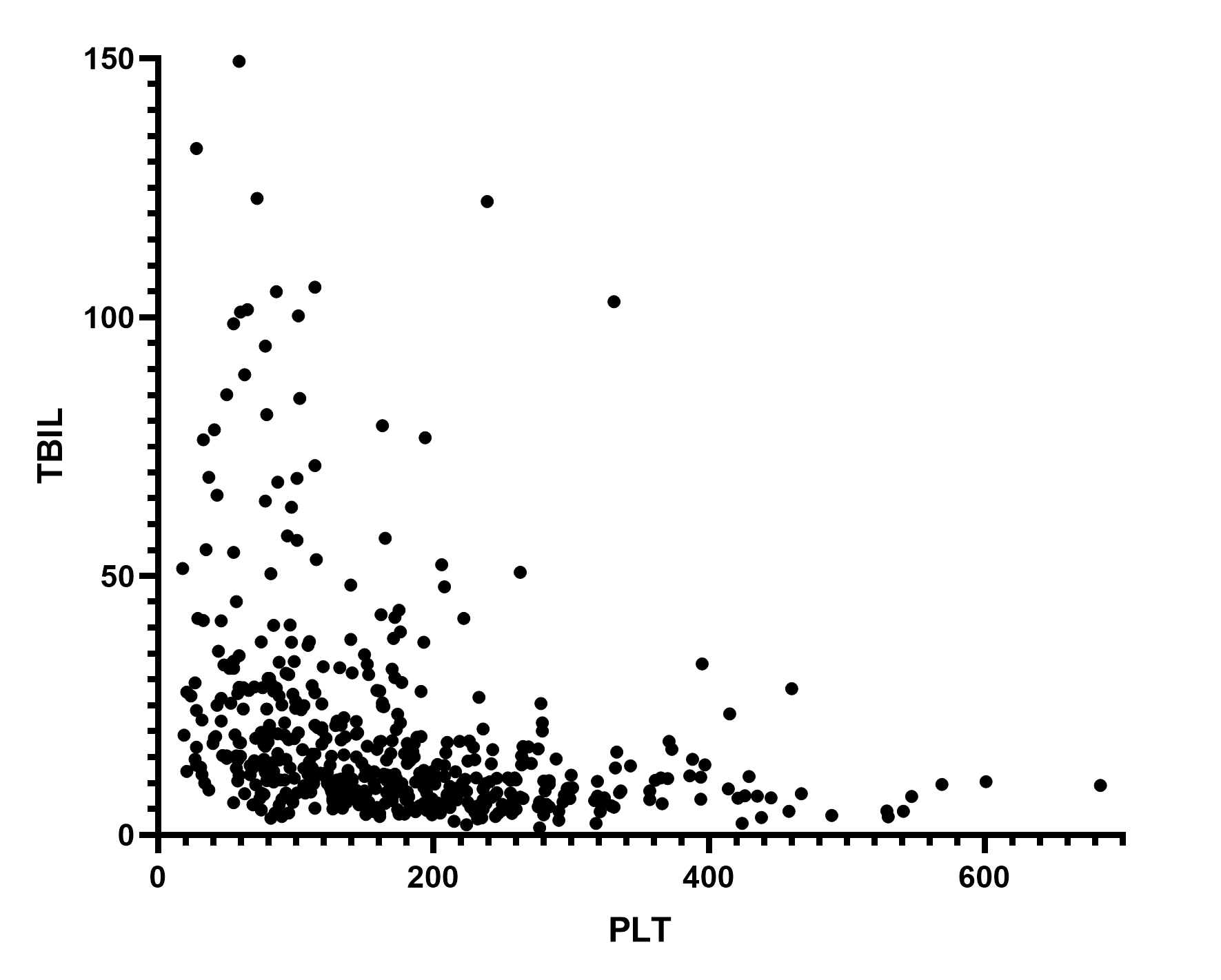

Supplement: Supplementary file 1 [file pharmaceuticals-17-00665-s001.zip › Figure S1B.png]

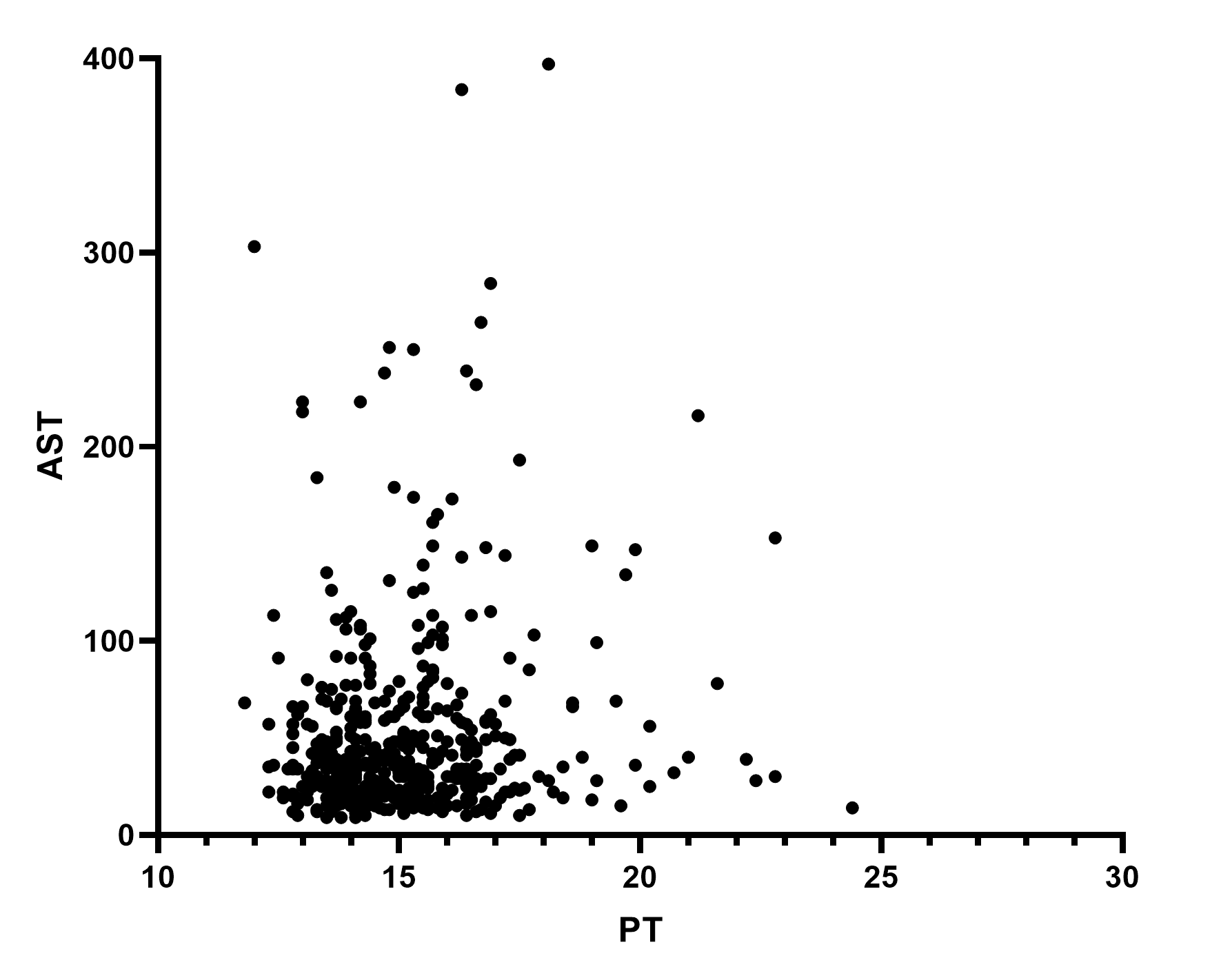

Supplement: Supplementary file 1 [file pharmaceuticals-17-00665-s001.zip › Figure S2A.png]

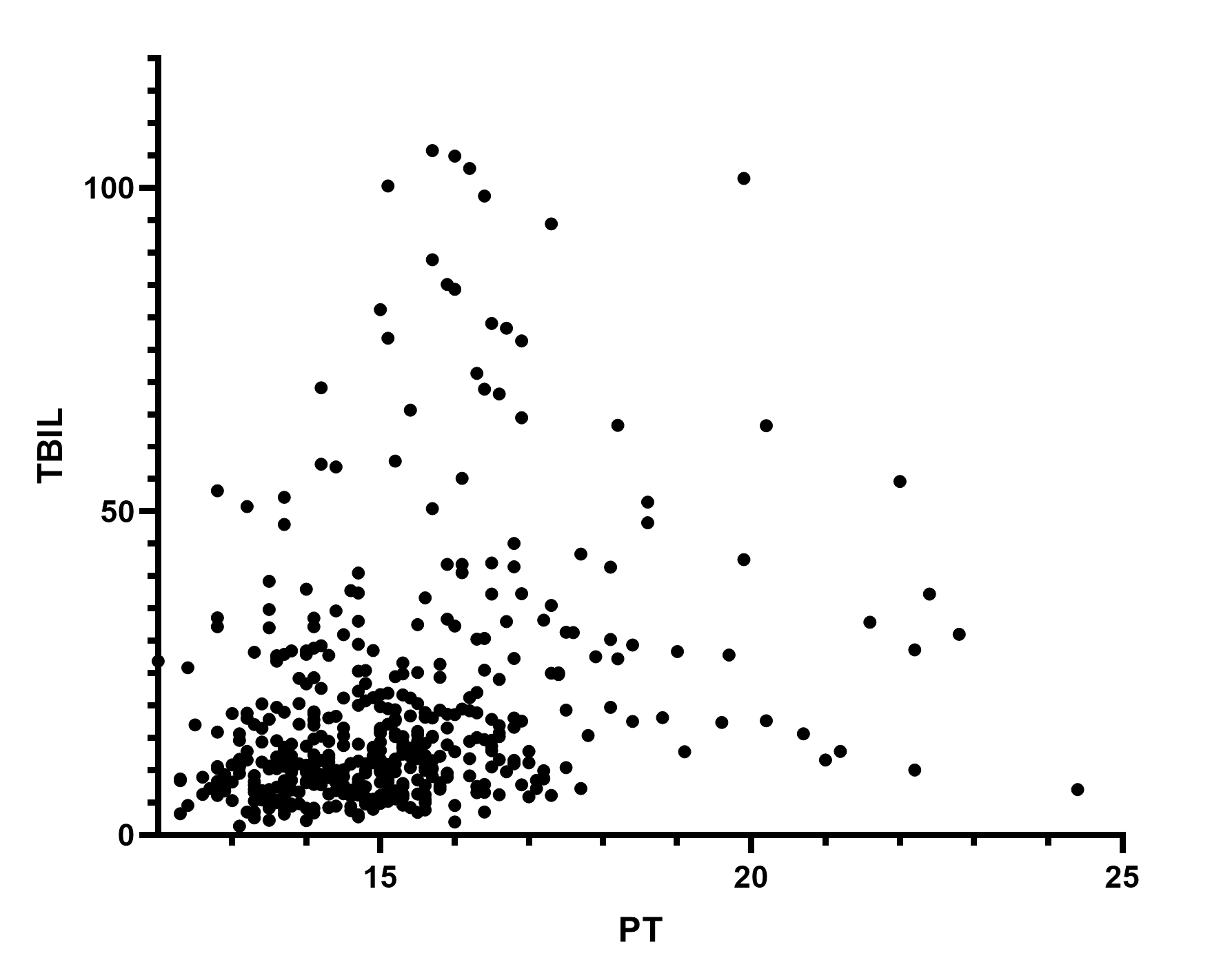

Supplement: Supplementary file 1 [file pharmaceuticals-17-00665-s001.zip › Figure S2B.png]
